# Smart City Data Sensing during COVID-19: Public Reaction to Accelerating Digital Transformation

**DOI:** 10.3390/s21123965

**Published:** 2021-06-08

**Authors:** Alexander A. Kharlamov, Aleksei N. Raskhodchikov, Maria Pilgun

**Affiliations:** 1 Institute of Higher Nervous Activity and Neurophysiology, Russian Academy of Sciences, 117865 Moscow, Russia; kharlamov@analyst.ru; 2 Moscow Centre of Urban Studies, 115280 Moscow, Russia; silaslowa@mail.ru; 3 Institute of Linguistics, Russian Academy of Sciences, 125009 Moscow, Russia

**Keywords:** smart city, social media, neural network technologies, speech perception

## Abstract

The article presents the results of the analysis of the adaptation of metropolis IT technologies to solve operational problems in extreme conditions during the COVID-19 pandemic. The material for the study was Russian-language data from social networks, microblogging, blogs, instant messengers, forums, reviews, video hosting services, thematic portals, online media, print media and TV related to the first wave of the COVID-19 pandemic in Russia. The data were collected between 1 March 2020 and 1 June 2020. The database size includes 85,493,717 characters. To analyze the content of social media, a multimodal approach was used involving neural network technologies, text analysis, sentiment-analysis and analysis of lexical associations. The transformation of old digital services and applications, as well as the emergence of new ones were analyzed in terms of the perception of digital communications by actors.

## 1. Introduction

The digital services, measurement and data management technologies of smart cities were stimulated to develop rapidly during the COVID-19 pandemic,. The public response to technological innovations and the introduction of new regulations was mixed. Some innovations were implemented quite successfully and were popular with the urban residents. Others have prompted various forms of resistance, from social media criticism to civil disobedience. This article is devoted to the analysis of the reaction of Moscow residents to accelerating digital transformation, which the Moscow authorities were forced to carry out during the onset of the COVID-19 pandemic.

There is already a sufficient amount of scientific research revealing various aspects of the use of IT technologies to combat the spread of COVID-19, accelerate digital transformation, as well as a study that analyzed the role of old and new media during the coronavirus pandemic, reactions on social networks and the transformation of communication processes, etc.

For example, researchers are actively developing technological solutions for detecting coronavirus, monitoring symptoms [1,2,3,4], rehabilitation [5], limiting the spread of the pandemic [6].

It should be noted that the “digital turn” radically changed politics, transformed the traditional modernist binary systems in state-society, public-private, consumption-production, work-leisure, culture-nature and man-posthuman terms. The growing influence of digital technologies on society has formed two opposite positions. Digital optimists focus on the positive aspects of digital transformations that provide new opportunities for the formation of diverse forms of communities, alternative ways of learning and perception, creative innovations, a culture of participation, networking and cloud democracy (“e-democracy”). Digital pessimists point to the negative aspects associated with the expansion of dominance through new forms of control, a surveillance society, network authoritarianism, digital dehumanization, alienation 2.0 and network exploitation. Socially disadvantaged people living on the “outskirts” of the digital society are oppressed both in terms of access to the benefits of use (there are three levels of the digital divide) and in terms of understanding and handling new digital technologies (three levels of separation) [7].

Research has shown that the majority of residents highly appreciated the benefits of using digital technologies in the field of transport and services provision; however, decision-making in the field of healthcare and justice involving Artificial Intelligence (AI) technologies causes a negative attitude. Residents’ greatest concerns are related to ethical issues, lack of transparency and the potential AI impact on employment. It is appropriate to recall that, according to the research by Carrasco, Mills, Whybrew and Jura, the level of confidence in the government was the decisive factor in the readiness of residents to master artificial intelligence technologies [8].

The growth of technology has led to the emergence of the concepts of “digital twin” and “digital identity” [9,10]. Digital twin technologies have proven to be extremely in demand in the formation and development of the smart city concept, since they provided ample opportunities for ensuring the safety and sustainable development of smart cities.

Researchers have proven that digital twin technologies used to monitor, visualize, diagnose and predict in real time are vital to the sustainability and efficiency of urban systems and infrastructure elements that interact with each other. Moreover, one of the key factors ensuring the security of digital twins is the Internet of things. The negative side of the development of digital technologies, as is recognized, are the risks associated with the loss of privacy of a person’s identity, since digital footprints provide unprecedentedly detailed information about private life. The privacy of a person is directly dependent on the effectiveness of his personal data protection [11].

Thus, with the development of “smart” cities and technologies that use various types of data, ethical problems are exacerbated that are associated with privacy, the possibilities of the Internet of things, “smart” infrastructure, digital government, etc.

Face recognition technologies are the most in demand in the “smart” city system. Mass video surveillance is designed to provide security and reduce the crime rate, but it does not correlate well with privacy and significantly expands the possibilities of control and surveillance. The Internet of things, on the one hand, makes it possible based on data to increase the efficiency of management decisions fight against crime and to increase the comfort of residents by creating new services; on the other hand, the data collected by Internet of things technologies is by no means limited to information about the state of urban infrastructure, but also contain personal data, as well as personal information of residents. As a result, fundamentally new risks for the safety of the city are formed; the urban infrastructure depends heavily on the manufacturers of technologies, software and components; there is a risk of data leakage.

The rapid development of artificial intelligence technologies in the near future will significantly expand the boundaries of digital technologies and will pose a number of new problems in terms of contradictions between the public good and the boundaries of a person’s private life in a “smart” city. Thus, decision support systems using artificial intelligence technologies can improve the efficiency of the decision support system and predictive analytics, organize preventive targeted social assistance, ensure a quick reaction of city authorities to changes, but increase the risks of discrimination due to the bias of algorithms and lead to an increase in digital inequality [12,13,14,15].

In the world, already in the pre-COVID-19 era, there were precedents of confrontation between city residents and technology campaigns. Thus, residents of the city of Toronto (Canada) refused the opportunity to turn their city into a “smart” one with the participation of the Alphabet Corporation because of the negative attitude of the residents towards data-corporations that commit gross violations in the collection and use of people’s personal data [16].

In the Netherlands, the contradictory nature of the processes accompanying the development of a “smart” city has led to the formation of the basic principles of urban planning that define the boundaries of the technology invasion into the residents’ lives [17].

Analysis of the development of epidemics in past years has shown that an increase in social tension is caused by communicative errors, which are made in the course of an information campaign on the prevention and treatment of the population [18,19,20]. Moreover, the main role in the conflict escalation and the growth of negative sentiments, is played by the media. The health care crisis causes panic and forms the collective anger in society that falls upon the authorities [21].

The results of previous studies were also confirmed by the practice of the COVID-19 pandemic, which showed that prevention and protection against infection in communities play a decisive role in containing and controlling the spread of infection [22].

Anti-crisis communications during the COVID-19 pandemic have already been described in detail in the specialized literature [23,24]. In particular, the study [25] analyzed the role of old and new media during the COVID-19 pandemic, reactions in social media, information support of quarantine measures, the phenomenon of infodemic and the transformation of communication processes.

The features of organizational communications during the pandemic [26], the transformation of the event management industry [27], examples of successful and unsuccessful leadership and guidelines for responding to COVID-19 [28] have also received attention.

The public reaction, which is expressed in the digital footprints of users, is usually analyzed using sentiment analysis. Modern sentiment analysis of the text includes at least three types of tasks: (1) classification of tonal messages (positive/negative or finer gradation); (2) determination of sentiment regarding a given sentiment object (often followed by visual marking of the sentence dependency tree; (3) determination of sentiment of a sentiment object with respect to its implicit and explicit attributes (feature-based) [29,30,31].

Sentiment analysis on the impact of coronavirus in social life using the BERT model is presented in a detailed study. [32]. However, it should be noted that the analysis of public sentiment based on digital data during the period of the pandemic was carried out mainly on the basis of tweets [33,34,35,36]. For Russian-speaking users, the analysis of Twitter content is not indicative, since this resource is not popular with users due to the morphological and syntactic features of the Russian language. In the Russian-speaking media space, Twitter is used primarily by PR experts and spin doctors in political communications. A technology for monitoring the mental health of citizens during the COVID-19 pandemic using sentiment analysis based on the material of the Korean language was proposed in the study [37]. Meanwhile, highlighting four emotional labels (anger, sadness, neutral, and happiness) and three possible interactive responses for each emotion (reciting wise sayings, playing music, and sympathizing: reciting wise sayings, playing music, and sympathizing) that gives good results for the Korean language users is not relevant for the analysis of the content of Russian-speaking users.

The novelty of this study lies in the fact that, despite the existing research, the specificity of the data analysis and management of the smart city during COVID-19 in terms of public reaction to the acceleration of digital transformation and the perception of digital innovations by residents has not yet received coverage in the specialized literature.

The aim of the study is to analyze actors’ perceptions of the accelerations of digital transformation, adaptation of IT technologies of the metropolis to solve operational problems in the extreme conditions of the first wave of the COVID-19 pandemic, as well as study society’s reaction to technical transformations, the specifics of analysis and management of urban infrastructure data.

The significance of the proposed work in the current situation is due to the fact that the spread of COVID-19 continues and acceleration of the digital transformation will also have to continue. Meanwhile, the success of digital transformation largely depends on the reaction of society. The algorithm proposed by the authors of this article can be used to analyze the reaction of society to different types of transformations, for predictive analytics of conflicts, to increase the level of trust and the effectiveness of dialogue between society and the government.

## 2. Materials and Methods

### 2.1. Data

The material for the study was data from social networks, microblogging, blogs, instant messengers, forums, reviews, video hosting services, thematic portals, online media, print media and TV related to the first wave of the COVID-19 pandemic in Russia.

Data collection was carried out using monitoring by message texts, recognized texts in pictures, video transcripts, check-ins, and stories. When forming the empirical database, various types of digital sources were used: social networks, blogs, forums, reviews, marketplaces, map services, stores of mobile applications; Telegram public channels and chats; online media; websites of government agencies, market-forming companies and organizations.

Data collection period: 1 March 2020 to 1 June 2020.
Database volume: 11,120,287 words and 85,493,717 characters.
Number of messages: 161,541.
Number of active actors: 47,574.
Number of sources: 1325.

### 2.2. Method

To analyze the content of social media, a multimodal approach was used involving neural network technologies, text analysis, sentiment-analysis, analysis of lexical associations [38] and content analysis [39,40]

The study involved a model using neural-like elements with temporal summation of signals or corticomorphic associative memory, which made it possible to single out explicit knowledge, topics that aroused the greatest interest of actors, to study the topic structure of content and to summarize data. In addition, the neural network representation of the text made it possible to form and interpret the semantic network in the form of a set of interrelated concepts. With the help of the semantic network, implicatures and semantic accents, which are most important for the actors, were analyzed and then rated. The analysis of associative networks of relevant stimuli made it possible to draw conclusions about the perception.

### 2.3. Procedures

1. Content selection and cleaning (filtering).

Data processing was carried out using the method of random Markov fields and its modification—the method of Conditional Random Fields (CRF). The CRF method, like the Maximum Entropy Markov Models (MEMM) method, refers to discriminative probabilistic methods, in contrast to generative methods such as Hidden Markov Models (HMM) [41] or the naive Bayes method [42]. By analogy with MEMM [43], the choice of factor-signs for setting the probability of transition between states in the presence of an observed value of xt depends on the specifics of specific data, but unlike the same MEMM, CRF can take into account any peculiarities and interdependencies in the initial data. The feature vector L = {λk} is calculated based on the training sample and determines the weight of each potential function. For training and application of the model, algorithms similar to those of HMM are used: Viterbi and its variant—the “forward–backward” algorithm [43,44]. It is believed that the CRF method is the most popular and accurate way of extracting objects from text and can be a significant competitor to other statistical methods used in linguistic text processing [45]. For example, it was implemented in the Stanford Named Entity Recognizer project [46].

1.1. Isolation and extraction of artificial entities (bots were carried out using a model using neural-like elements with temporal summation of signals.
1.2. Content clustering was performed using dynamic network metrics, trail metrics, procedures for grouping nodes, identifying local patterns, comparing and contrasting networks, groups, and individuals from a dynamic meta-network perspective

2. Performing sentiment analysis.

In this study, sentiment analysis was performed using the Eureka Engine sentiment determination module. The technique is based on a statistical algorithm for conditional random CRF fields using sentiment dictionaries. Sequences of lexems are used as input data, after which the algorithm calculates the probabilities of possible sequences of tags and chooses the maximum probable one.

3. Performing Content Analysis.

Content analysis was performed in accordance with [39,40] using the AutoMap text mining tool.

4. Identifying key topics.

The procedures listed in paragraphs 4–6 were carried out avail of a model using neural-like elements with temporal summation of signals or corticomorphic associative memory, which made it possible to single out explicit information [38].

4.1. Selection and analysis of the topic structure.
4.2. Summarization.

5. Constructing a semantic network
  5.1. Extraction of the semantic core (nominations with link weights of 98–100).
  5.2. Textual analysis of the semantic core.
6. Analysis of associative network.
  6.1. Performing an associative search.
  6.2. Word associations

### 2.4. Tools

To collect data, the Brand Analytics (https://br-analytics.ru/) (accessed on 30 January 2020) and Sketch Engine (https://www.sketchengine.eu/) systems were used.

The verbal content was analyzed using the neural network technology TextAnalyst 2.3. (http://www.analyst.ru/index.php?lang=eng&dir=content/products/&id=ta) (accessed on 30 January 2020) developed by one of the article authors, A.A. Kharlamov.

ORA-LITE was used for network analysis, which is a dynamic meta-network assessment and analysis tool specifically developed (http://www.casos.cs.cmu.edu/projects/ora/) (accessed on 30 January 2020)

Content analysis was performed using the AutoMap text mining tool (http://www.casos.cs.cmu.edu/projects/automap/) (accessed on 30 January 2020).

For visual analytics, the Tableau platform was used (https://www.tableau.com/) (accessed on 30 January 2020).

## 3. Results

### 3.1. General Description of the Content

The period of the pandemic onset in Russia was selected for the study. The database was divided by type of actors into two groups by geolocation. Since the spread of the coronavirus infection in Russia began from the capital, the content was divided into two groups to ensure correct analysis: Moscow actors and actors from the Moscow region, other regions of Russia, as well as Russian-speaking actors from other countries, which were conditionally designated as regional.

**Figure 1:**
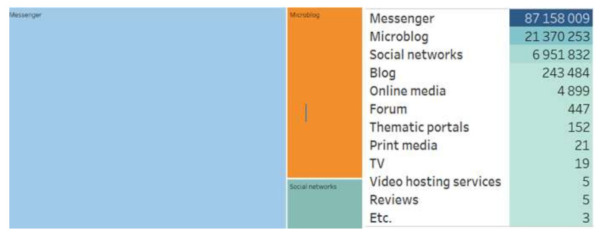
Digital platforms of regional actors.

**Figure 2:**
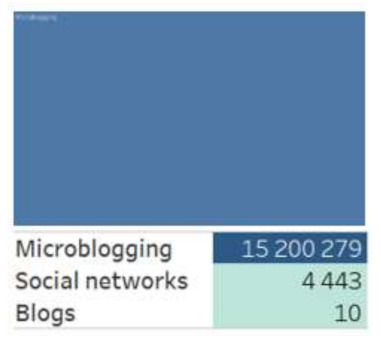
Digital platforms of Moscow actors.

Both groups of actors differ in their preferences for the digital platforms they have chosen to generate content on the coronavirus infection spread. The group of regional actors is more diverse, they used mostly messengers; the Moscow community preferred microblogging (Figure 1 and Figure 2).

Analysis of the data shows a difference in the development and perception of the coronavirus topic among Moscow and regional actors. Moscow actors began discussing COVID-19 earlier and with greater intensity. The activity index of Moscow actors expressed in the number of posts per author (11.69) is 3.5-times higher than the regional index (3.33).

### 3.2. Content Sentiment Analysis

**Figure 3:**
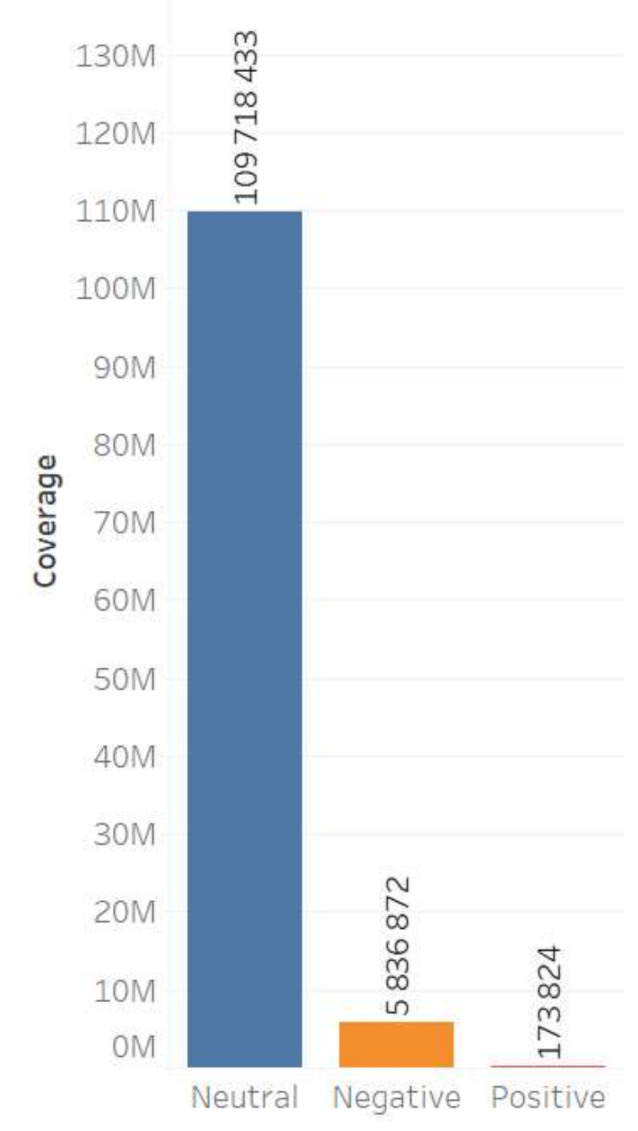
Sentiment of the regional actors’ content.

**Figure 4:**
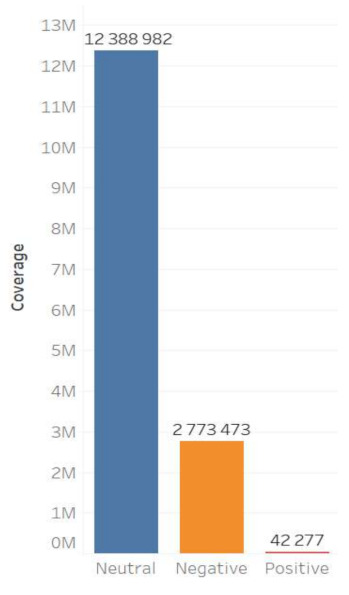
Sentiment of the Moscow actors’ content.

The content of Moscow actors is also characterized by a higher degree of negative reactions. It should be noted that the neutral cluster predominates in the database for both groups, while in the Moscow content the negative cluster is more extensive, and the positive one is nearly non-existent (Figure 3 and Figure 4).

**Figure 5:**
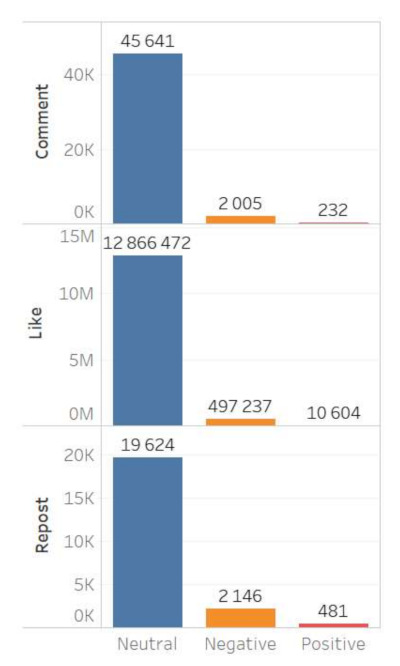
Sentiment of digital footprints of the regional actors’ content.

**Figure 6:**
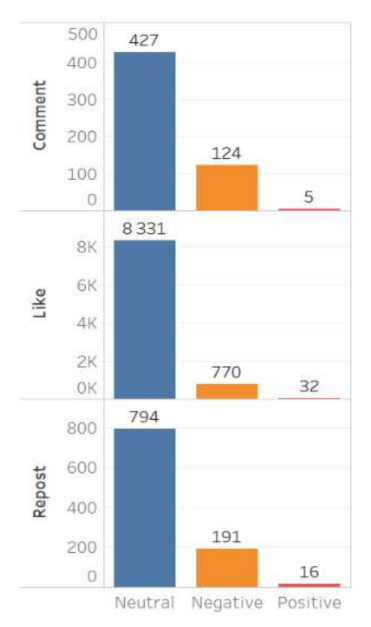
Sentiment of digital footprints of the Moscow actors’ content.

The sentiment features of digital footprints also indicate a greater intensity of negative emotions in the Moscow group: 29.03% of negative comments; and in the regional group only 4.39% of comments are negative (Figure 5 and Figure 6).

### 3.3. Key Topics of the Content

Identification and analysis of the topic structure, summarization and content analysis made it possible to determine that the key topics of the regional content related to the pandemic in this period were as follows:

- Emergence of coronavirus infection in Moscow;
- Coronavirus spread in the Moscow region;
- Pandemic spread throughout Russia;
- Discussion of the specifics of the new disease (Appendix A).

In the Moscow content, the topics were more diverse:

- Discussion of the measures taken to fight the infection spread;
- Health problems that have worsened since the beginning of the quarantine;
- Criticism of the actions of the authorities regarding the fight against the coronavirus infection;
- Political issues;
- Discussion of constitutional amendments (Appendix B).

### 3.4. Core of the Semantic Network

The semantic network makes it possible to identify semantic accents that are the most significant for actors, to analyze the implicit knowledge hidden behind explicitly expressed speech structures. The core of the semantic network was identified from the nominations with link weights of 98–199.

**Figure 7:**
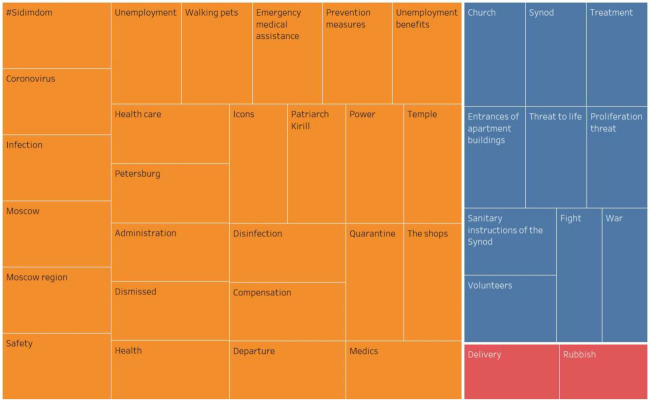
Core of the semantic network of the regional actors’ content.

The regional actors pay special attention to the threat of the coronavirus spread, medical problems and anti-coronavirus measures. The regional content distinguishes the discussion of the negative economic consequences of the pandemic (*unemployment*), as well as Orthodox topics (*Patriarch Kirill, Russian Orthodox Church (ROC), Synod, Sanitary Instruction of the Synod, Church*). In addition to *Moscow*, the *cities of the Moscow Region* and *St. Petersburg*, in the semantic network of the regional authors, the nominations of the following Russian cities have large link weights: *Barnaul, Bataysk, Blagoveshchensk, Bugulma, Vladivostok, Volgograd, Volzhsky, Vologda, Vlasovo, Grigorievsk, Irkutsk, Kaluga, Kamensk-Uralsk, Kemerovo, Kirov, Kovrov, Kronshtadt, Lipetsk, Naberezhnye Chelny, Novokuznetsk, Omsk, St. Petersburg, Surgut, Taganrog, Togliatti, Tomsk, Tyumen*, etc. (Figure 7) (colors and sizes of shapes represent three clusters according to the weight of the vertices).

**Figure 8:**
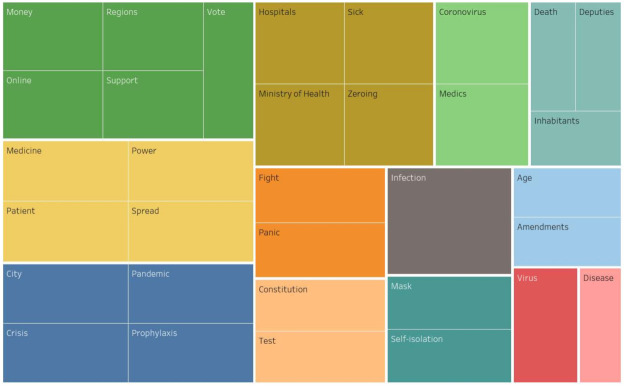
Core of the semantic network of the Moscow actors’ content.

In the core of the semantic network of the Moscow actors, semantic accents were identified related to the spread of the pandemic and its prevention. Muscovites are also worried about the state of medicine, the specifics of treatment, the availability of drugs, the state of hospitals, the efficiency of the Ministry of Health, the number of deaths, as well as the economic crisis and the actions of the authorities. In addition to the problems of the coronavirus infection spread, political (*zeroing, amendments, Constitution, voting, rights*) and economic issues are of great importance for the Moscow actors. It is significant that in the Moscow content there are no mentions of Russian cities at all, except for *Moscow*, but there are the following nominations: *regions, Italy, China, Europe, USA* (Figure 8).

### 3.5. Associative Network

The analysis of lexical associations was performed according to the results of the associative search and the construction of an associative network made it possible to identify implicatures and subtext information characterizing the attitude of the actors to certain processes and phenomena.

**Figure 9:**
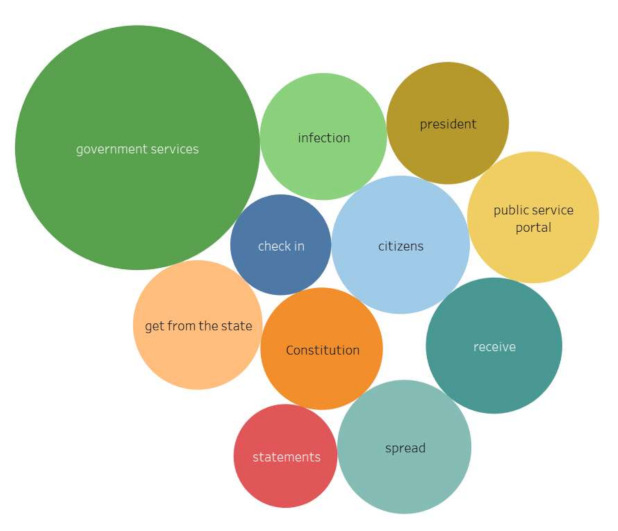
Associative network for stimulus *state* (10/24,393), regional actors).

**Figure 10:**
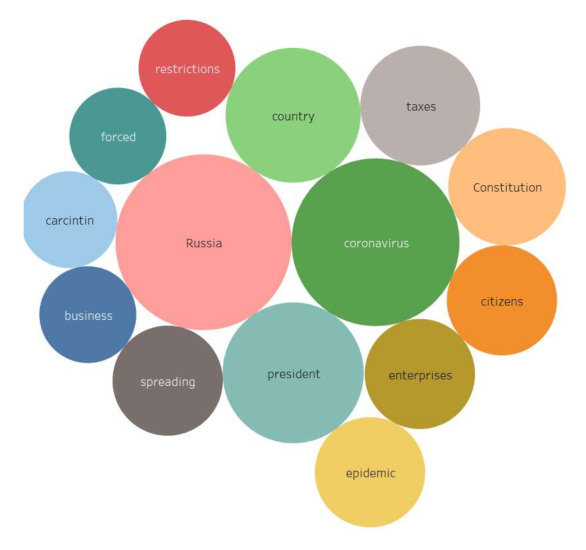
Associative network for stimulus *state* (10/2,510), Moscow actors).

**Figure 11:**
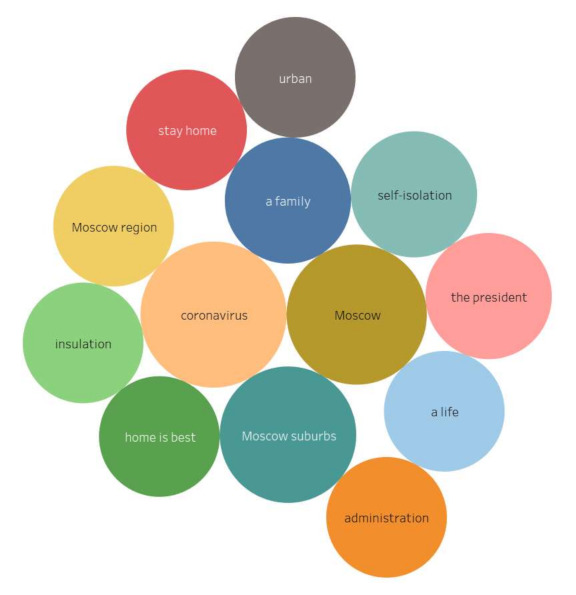
Associative network for stimulus *authority* (10/10,946, regional actors).

**Figure 12:**
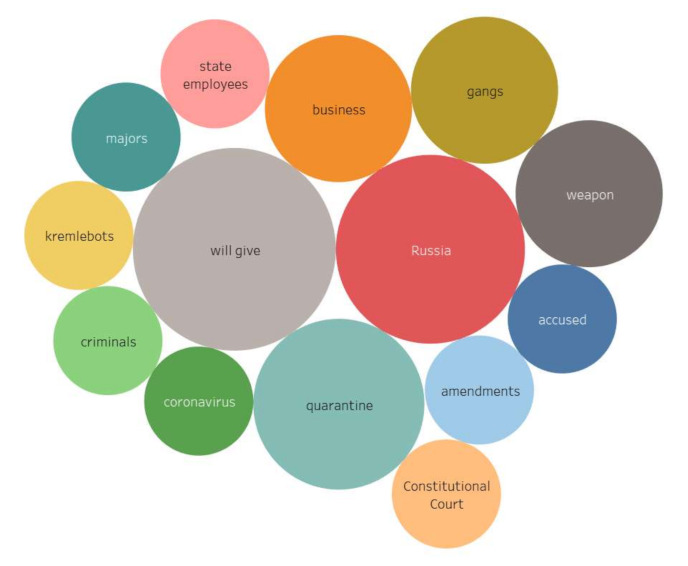
Associative network for stimulus *authority* (10/24,393, Moscow actors).

The data analysis revealed that Russian-speaking actors pinned their greatest hopes on government bodies during the crisis caused by the coronavirus infection spread. Meanwhile, it should be noted that a group of Moscow actors stands out among the digital communities that showed sharp negative reactions. Thus, the stimulus *authority* in the content of the Moscow actors has the following reactions: *kremlinbots, majors, accused, amendments, criminals, gangs, weapons*. The stimulus *state* does not cause negative reactions in both groups, as well as stimulus *authority* among the regional actors (Figure 9, Figure 10, Figure 11 and Figure 12) (the size and color of the circle depends on the weights of the vertices).

**Figure 13:**
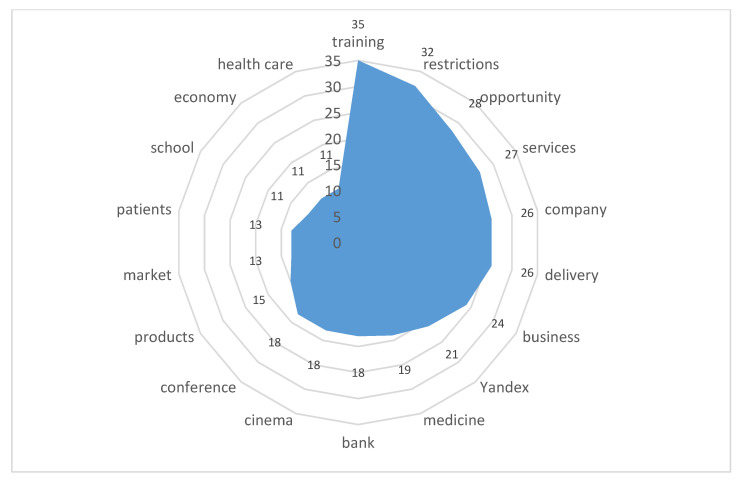
Associative network for stimulus *online-services.*

The attitude towards the strengthening of digital transformation is almost identical in all digital communities, therefore, below are the reactions derived from the common database:

- Stimulus *online-services* (10/99,460) (Figure 13)

The highest index of 10/99,460 among digital transformations was received by online services and applications, which the actors discussed most often and intensively. It was these technological solutions that helped the city residents cope with the conditions of movement restriction and the danger of infection. The most in demand were delivery services, medical and banking services, home digital theatres, applications for schoolchildren and platforms for convening conferences. Among the IT companies that provide such services, the actors distinguish the Russian holding Yandex.

The content included the following reactions:

“(…) Russians started buying groceries more often in “neighborhood stores”: the indicator there grew by 10%; on the contrary, in large supermarkets, the volume of expenditures fell by 3%. VTB also notes an increase in expenses for home delivery of groceries and home online services (…).”

**Figure 14:**
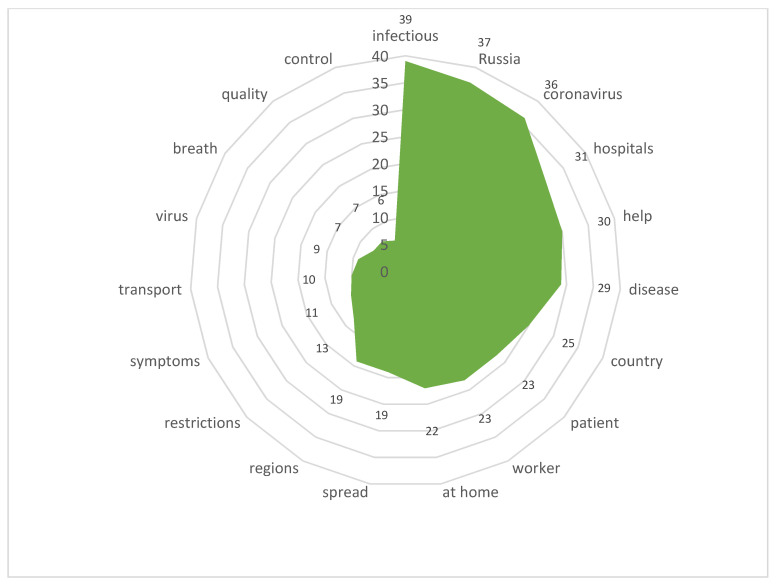
Associative network for stimulus *online-medicine.*

- Stimulus *online-medicine* (10/84,101) (Figure 14)

The natural high interest of the actors was aroused by the possibilities of online-medicine (the stimulus index is 10/84,101) during the pandemic. Consulting services involving digital resources at the stages of prevention, diagnosis, rehabilitation and treatment of mild forms of the disease facilitated the organization of individual medical care and a reduced burden on medical personnel to a certain extent. It should be noted that this situation is typical mainly for the city of Moscow. In the regions, the pandemic revealed the shortcomings of digital development.

The content included the following reactions:

“During the Ebola epidemic in West Africa in 2014–2016, more people died from disruptions to daily health care than from the disease. Telemedicine should become more accessible and people with chronic conditions should receive medicines for three months whenever possible, in case of supply disruptions (...) preventive services should be continued.”

**Figure 15:**
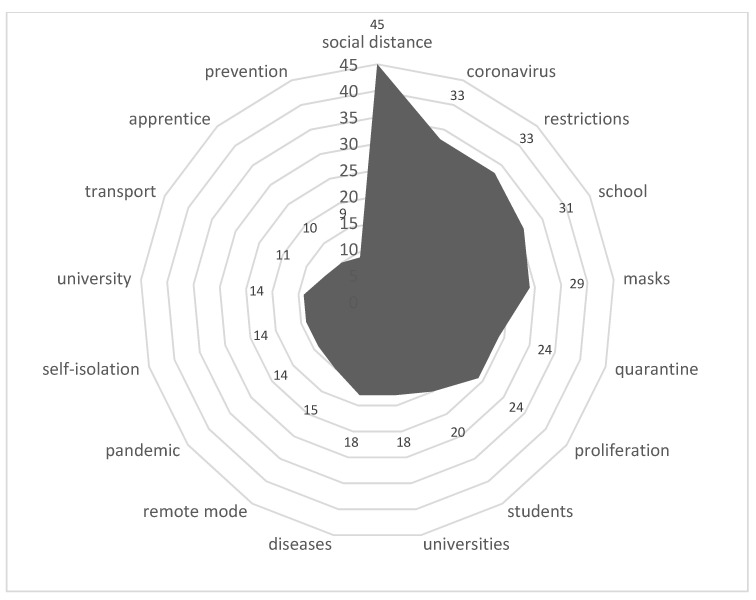
Associative network for stimulus *distance learning.*

- Stimulus *distance learning* (10/77,131) (Figure 15)

The need to transfer the learning process to the distance learning form caused a strong reaction, especially in secondary schools, from both teachers and parents. The technical unreadiness of schools and staff and the lack of teaching materials led to a very difficult situation at the beginning of the transition to the online format. Later, the situation stabilized somewhat.

The content included the following reactions:

“Confusion. I can’t say it was just fear, because, as they say, it is necessary. But how to do it? How should it look like, how should all this be organized? These questions were considered, of course.”

**Figure 16:**
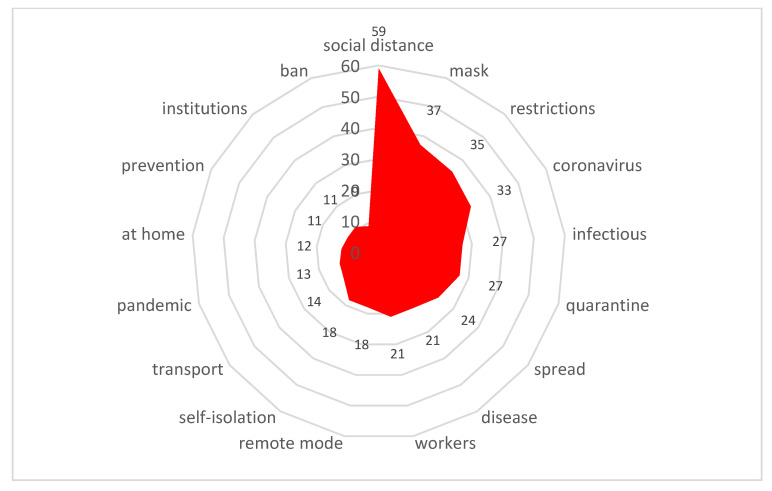
Associative network for stimulus *distance work.*

- Stimulus *distance work* (10/75,473) (Figure 16)

The transition to distance working in a metropolis has led to the need to stay in a small enclosed space for all family members. The inability to go outside during the quarantine was especially painful for families with small children. As a result, there has been an unprecedented migration from metropolises to suburbs and small towns.

The content included the following reactions:

“Due to the COVID-19 pandemic, many office workers have switched over to remote work and have found that they have more stress and less time for themselves. It turned out that when they took a couple of hours every day to get to and from work, there was more freedom. The number of excessive work hours are now three hours a day on average.”

**Figure 17:**
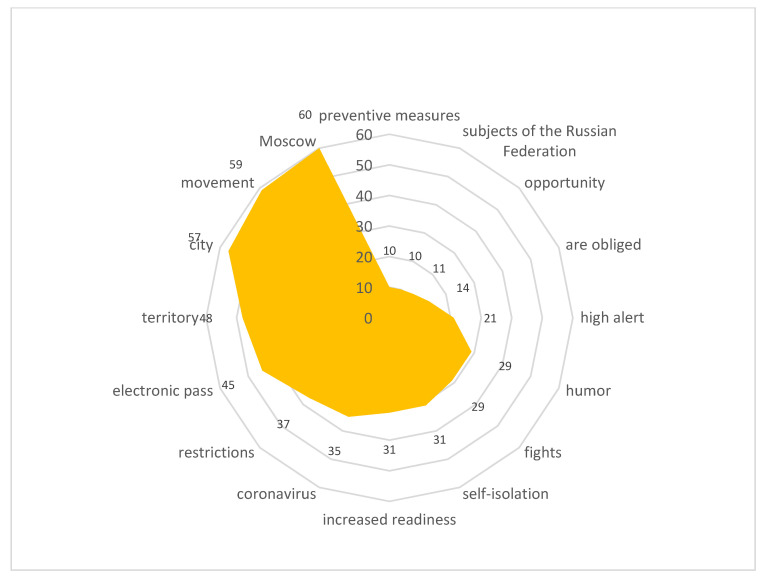
Associative network for stimulus *digital pass.*

- Stimulus *digital pass* (10/66,653) (Figure 17)

The introduction of digital or electronic passes to get around the city provoked a violent reaction from the actors. It should be noted that some of the city residents showed understanding toward this innovation as a means to stop the spread of infection. Others began to actively discuss the violation of the residents’ rights and freedoms. Negative reactions were fueled by technological failures that led to the automatic issuance of fines for people who passed the registration correctly or did not leave their apartments at all.

The content included the following reactions:

“In Moscow, electronic passes based on 16-digit alphanumeric combinations were introduced, the issuance of which began on 13 April, and on 14 April it became known about the cancellation of almost a million of them: the reason was incorrect or inaccurate data indicated during their registration.”

**Figure 18:**
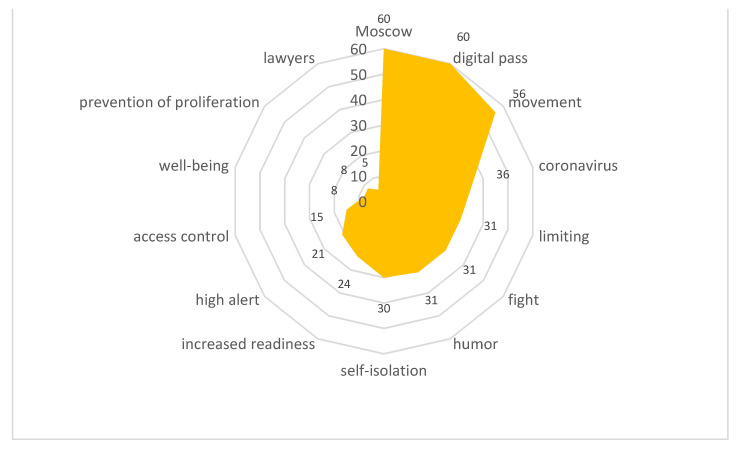
Associative network for stimulus *electronic pass.*

- Stimulus *electronic pass* (10/63,726) (Figure 18)

The content included the following reactions:

“Since nothing makes a person so desperate that he/she no longer believes in anything holy and bright, as the inability to leave the house and go somewhere, by car, subway, bus, and sometimes just walk down the street or take a walk in the park. For example, in Russia, the mayor of Moscow is criticized a lot for what he did: with the onset of the viral madness, he introduced various restrictions for people who could not just go out into the street. And the electronic passes, without which it was impossible to get on any city transport, were called “the beginning of the end of the world.”

**Figure 19:**
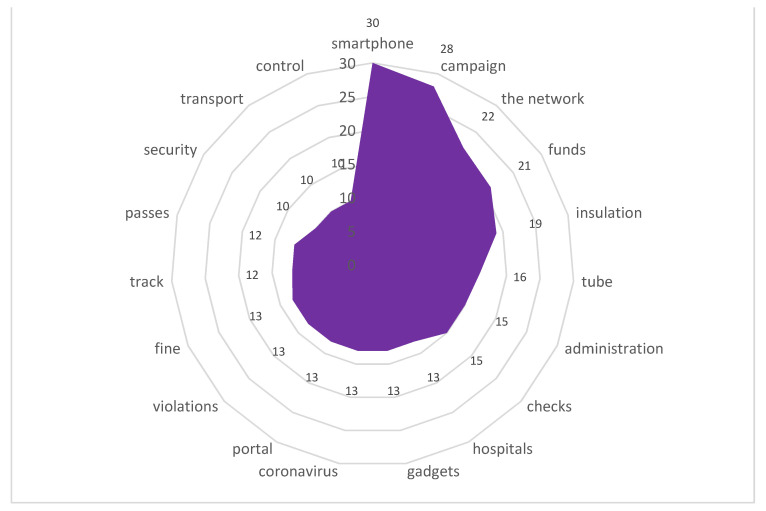
Associative network for stimulus *surveillance camera.*

- Stimulus *surveillance cameras* (10/35,468) (Figure 19)

Surveillance cameras have long been controversial in society. In August 2020, based on complaints from city residents concerning interference with their privacy, access to their biometric data and the technical security of such data in connection with the use of video cameras of the city video surveillance network with a face recognition system, RosKomSvoboda sent an official request to the Information Technology Department of the city of Moscow and the Ministry of Internal Affairs of Russia in Moscow. Later it was discovered that data from video surveillance cameras were sold on the Darknet; law enforcement officers tracked down two malefactors who turned out to be employees of the Ministry of Internal Affairs, and a criminal case was opened against them (https://roskomsvoboda.org/post/court-fined-police-for-leak-of-biometric-data/) (accessed on 30 January 2020). Supporters argued that ubiquitous video surveillance makes it possible to maintain safety in an urban environment, while opponents insisted there was a violation of privacy as city residents found themselves defenseless against data leaks and sales, the collection of redundant data for profit, non-compliance or formal compliance with the law, and ineffective protection or unwillingness to protect personal data.

It is significant that during the quarantine period, data from surveillance cameras were used by both government agencies to detect violators of quarantine measures, and city residents to prove illegal actions, for example, of law enforcement agencies.

The content included the following reactions:

“The Investigative Committee opened two criminal cases. Alexander Konovalov, in turn, took the offensive and published two posts at once on his social networks. On the first, he showed how the security-service agents knock down the door of a restaurant and put everyone on the floor. The footage shows that some of those present in the bar took a beating with batons from the security-service agents in masks (it is unclear whether they are visitors or employees of the restaurant). The surveillance camera footage is accompanied with an emotional post.”

**Figure 20:**
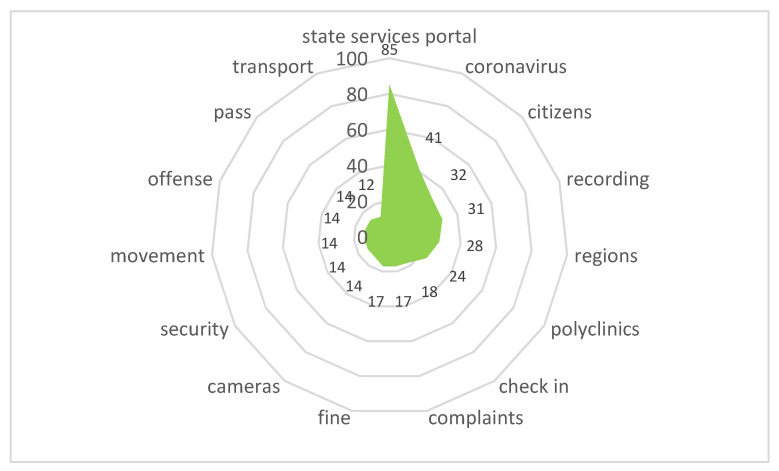
Associative network for stimulus *Government Services.*

- Stimulus *Government Services* (10/32,509) (Figure 20)

During the period of quarantine and self-isolation, portals for the provision of electronic government services, court websites, etc. made it not possible to stop proceedings for important civil issues. Meanwhile, technical failures that occurred due to the increased load caused a negative assessment from city residents.

The content included the following reactions:

“The first exultation regarding the fact that the authorities finally turned around to face their citizens and began to respect their dignity and time, was replaced by disappointment and even indignation!”

## 4. Discussion

The crisis caused by the pandemic has led to the need to accelerate digital transformation, to find optimal solutions to overcome the negative consequences in various spheres of society. Thus, technologies that can help cope with the global threat, including drones to detect infected people, robots that replace and protect medical personnel working with infected people; and blockchain technologies to ensure the confidentiality of transactions are investigated in [47]. Artificial intelligence technologies have become in demand for predictive analytics, effective decision-making in healthcare [48] and the formation of epidemic forecasting models and control systems [49]. Intelligent systems are being actively implemented to prevent the coronavirus infection spread [50], to stop the pandemic and reduce risks [51]. To overcome the consequences of COVID-19, big data analysis tools and artificial intelligence methods are used [52], as well as technological solutions for data mining [53,54]. The concepts of nanotechnology in the fight against the COVID-19 pandemic are presented in [55].

It is clear that the pandemic has caused enormous damage to all aspects of society. Thus, in the real sector of the Russian economy, according to a study conducted by Google, the Center for Training Leaders and Teams of Digital Transformation (RANEPA) and the Center for Advanced Management Solutions, 33% of the total number of Russian companies in the first half of 2020 suffered losses of more than 1.5 billion rubles, 46% of representatives of business structures announced a decrease in demand for their products or services. In addition, 46% of the population noted a significant reduction in income, and 33% in savings.

Meanwhile, the pandemic had a stimulating effect on the development of digital technologies: 57% of business representatives noted the acceleration of digitalization within their companies, 38% noted changes in their management culture and corporate culture, and 29% noted a reduction and reorganization of ineffective components of the business process (departments, sections, regulations, etc.).

The pace of digitalization within the corporate and public sector has increased, as well as the digitalization of processes that are less effective in the “analog” form. The introduction of digital transformation in government structures began to be perceived more positively, since responsible officials saw the real benefits of these transformations.

Society has also been forced to use digital techniques more actively. More than 33% of respondents aged 31 to 45 answered that they have started to use digital services more often. Wealthy and educated residents of metropolises are most positive about digital transformation, which implies expanding economic opportunities. Residents of small settlements express fears related to tax increases, job losses and reduced opportunities to find work in the “gray” area [56].

## 5. Conclusions

Analysis of data from Russian-speaking users showed that the intensification of digital transformation during the COVID-19 pandemic caused a controversial response from society.

As a result of the study, for the first time, materials were collected and analyzed that made it possible to determine the features of the analysis of data from a smart city, via the digital traces of residents during the onset of the COVID-19 pandemic. The city of Moscow was used as an example to analyze society’s response to the acceleration of digital transformation and its citizens’ perception of digital innovations.

The study confirmed the assumptions that the growth of social tension directly depends on the communication strategies and the tactics that government agencies choose to inform residents with during the pandemic. To prevent panic caused by the healthcare crisis and the formation of collective anger, it is necessary to establish a dialogue between the authorities and society, and to organize targeted and personal assistance that can be implemented using digital resources. Meanwhile, it should be borne in mind that technological failures of such services are extremely painful for society during the crisis period. It was also confirmed that the willingness of citizens to use new smart city technologies of directly depends on the level of trust in social institutions, authorities and technology corporations. In addition, the thesis was confirmed that prevention and protection from infection in communities play an important role in containing and controlling the spread of infection.

The most popular among actors were online services providing delivery, medical, banking services, home digital theatres, applications for schoolchildren and platforms for convening conferences. Among the IT companies that provide such services, the actors distinguish the Russian holding Yandex.

Distance learning, digital passes and surveillance cameras received the most negative reactions from actors. The need to transfer the learning process to the distance learning form caused a strong reaction, especially in secondary schools, both among teachers and students’ parents. Digital passes and surveillance cameras demonstrate how the benefits of digital technology can lead to risks of unethical use of personal data and privacy breaches. The main fears of the actors were related to fines for erroneous data from video cameras and transport related to movement around the city, as well as technological errors with passes.

Portals for the provision of electronic government services, court sites, etc., made it possible not to stop proceedings for important civil issues, but technical failures that occurred due to the increased load caused a negative assessment from city residents, especially in connection with wrongful fines.

The speed of coronavirus infection in Russia began from the capital, which explains the selection of actors of two groups in the database by type: Moscow and regional (actors from the Moscow region, other regions of Russia, as well as Russian-speaking actors from other countries). The analysis of the content showed the politicization, opposition and territorial egocentrism of the former, which is not observed among regional actors in the period under study. The reactions of Moscow users are characterized by negative perception and skepticism; regional actors are more loyal, showing more support and approval of the actions of the state structures during the first wave of the pandemic, in particular, regarding the strengthening of digital transformations.

Future directions of research may be related to identifying the reaction of society to various measures that the authorities are proposing to stop the pandemic, identifying the most effective resources for digital transformation.

## Data Availability

Publicly available datasets were analyzed in this study.

## Appendix A

Thematic structure of the content of regional actors:

## Appendix B

Thematic structure of the content of Moscow actors
